# Measuring telomere length for the early detection of precursor lesions of esophageal squamous cell carcinoma

**DOI:** 10.1186/1471-2407-13-578

**Published:** 2013-12-05

**Authors:** Shih-Wen Lin, Christian C Abnet, Neal D Freedman, Gwen Murphy, Rosana Risques, Donna Prunkard, Peter Rabinovitch, Qin-Jing Pan, Mark J Roth, Guo-Qing Wang, Wen-Qiang Wei, Ning Lu, Philip R Taylor, You-Lin Qiao, Sanford M Dawsey

**Affiliations:** 1 Division of Cancer Epidemiology & Genetics, National Cancer Institute, 9609 Medical Center Drive, Bethesda, MD 20892, USA; 2 Department of Pathology, University of Washington, 1959 NE Pacific Ave., Seattle, WA 98195, USA; 3 Cancer Institute, Chinese Academy of Medical Sciences, P. O. Box 2258, Beijing 100021, People’s Republic of China; 4 Laboratory of Pathology, Center for Cancer Research, National Cancer Institute, Building 10, Bethesda, MD 20892, USA

**Keywords:** Esophageal squamous cell carcinoma, Esophageal squamous dysplasia, Early detection, Screening, Balloon cytology, Telomeres

## Abstract

**Background:**

Esophageal cancer is the sixth leading cause of cancer death worldwide; current early detection screening tests are inadequate. Esophageal balloon cytology successfully retrieves exfoliated and scraped superficial esophageal epithelial cells, but cytologic reading of these cells has poor sensitivity and specificity for detecting esophageal squamous dysplasia (ESD), the precursor lesion of esophageal squamous cell carcinoma (ESCC). Measuring telomere length, a marker for chromosomal instability, may improve the utility of balloon cytology for detecting ESD and early ESCC.

**Methods:**

We examined balloon cytology specimens from 89 asymptomatic cases of ESD (37 low-grade and 52 high-grade) and 92 age- and sex-matched normal controls from an esophageal cancer early detection screening study. All subjects also underwent endoscopy and biopsy, and ESD was diagnosed histopathologically. DNA was extracted from the balloon cytology cells, and telomere length was measured by quantitative PCR. A receiver operating characteristic (ROC) curve was plotted for telomere length as a diagnostic marker for high-grade dysplasia.

**Results:**

Telomere lengths were comparable among the low- and high-grade dysplasia cases and controls, with means of 0.96, 0.96, and 0.92, respectively. The area under the ROC curve was 0.55 for telomere length as a diagnostic marker for high-grade dysplasia. Further adjustment for subject characteristics, including sex, age, smoking, drinking, hypertension, and body mass index did not improve the use of telomere length as a marker for ESD.

**Conclusions:**

Telomere length of esophageal balloon cytology cells was not associated with ESCC precursor lesions. Therefore, telomere length shows little promise as an early detection marker for ESCC in esophageal balloon samples.

## Background

Esophageal cancer is the 6^th^ leading cause of cancer death worldwide and was estimated to have killed 406,800 people in 2008 [1]. Over 80% of esophageal cancer cases and deaths occur in developing countries [1], and in these areas, 90% of these cases are esophageal squamous cell carcinoma (ESCC) [1,2]. Esophageal cancers can be successfully treated if diagnosed early [3], but tumors are usually asymptomatic until they reach an advanced stage, when they are much more difficult to cure. In the United States, the overall 5-year relative survival rate for esophageal cancer is 19% [4], but in low-resource populations, in which most esophageal cancer cases occur, the survival rate may be as low as 3% [5]. Asymptomatic patients with precursor lesions can be treated to prevent progression to invasive tumors and death [6,7], but current screening tests for precursor lesions are inadequate.

One of the highest risk regions for ESCC is in north-central China, which includes the county of Linxian [8]. Previous studies by our group in this region have shown that esophageal squamous dysplasia (ESD) is the clinically relevant precursor lesion of ESCC [9,10] and that ESD can be accurately identified with the use of Lugol’s iodine staining during endoscopy and confirmed with biopsy [11]. However, endoscopy is time-consuming, invasive, and also requires specially trained personnel and equipment to perform the examination, take biopsies, and make appropriate pathologic diagnoses, so frequent endoscopy for ESCC early detection screening in high-risk asymptomatic populations in underdeveloped settings with inadequate health resources remains a major challenge [12].

Balloon cytology, a simple and inexpensive method of retrieving esophageal cells, has been commonly used in China for diagnosing patients with dysphagia or for screening asymptomatic, high-risk populations for esophageal cancer [13]. In our previous studies, balloon cytology using traditional cytologic examination had poor sensitivity and specificity for detecting ESD [14,15]. Thus, if a biomarker of esophageal disease that could be measured in the balloon cytology cells could improve the sensitivity and specificity of this cell collection technique for detecting ESD, it might make an important public health impact. A validated early detection marker for ESD might also eventually serve as a target for the future development of inexpensive and rapid point-of-care molecular diagnostics that could be used to augment balloon cytology in resource-limited locations.

One hypothesized biomarker of neoplastic disease is telomere length. Telomeres are regions of repetitive nucleotide sequence at the ends of chromosomes that protect these ends from deterioration or fusion with neighboring chromosomes [16]. Chromosome replication during cell division results in telomere shortening. In the absence of the telomerase reverse transcriptase enzyme, which maintains telomere length, cells undergo replicative senescence. Thus, telomere length and the restriction of telomerase activity may play important roles in the prevention of uncontrolled cell division [17]. Telomere dysfunction or shortening is a common, and often early, genetic alteration acquired in a cancer [18]. Telomere length may serve as a marker of both chromosomal instability and cancer development [19].

Previous work has found that telomere length is associated with cancer incidence and mortality [20]. Several studies have examined the association between telomere length and neoplastic progression, including studies of biliary tract [21], colon [22-24], lung [25], and prostate [26] cancer. Telomere length abnormalities have been found to occur early in the initiation of epithelial carcinogenesis and may be an initiating event in many human epithelial cancers [27]. Shortened telomeres have been found in cancer cells isolated from paraffin-embedded sections of ESCC tumor biopsies [28]. Furthermore, patients who underwent esophageal resection as a result of ESCC had shorter telomeres in their tumors relative to their nearby non-neoplastic esophageal epithelial cells. However, it is particularly important to note that both the tumor and the nearby non-neoplastic esophageal epithelial cell types from cancer patients had shorter telomere lengths than the cells collected from non-cancer individuals with normal esophageal epithelium, suggesting a telomere-shortened epithelial field in the cancer patients [29]. In addition to studies in ESCC, some other studies have focused on individuals with Barrett’s esophagus, who are at increased risk of esophageal adenocarcinoma, another type of esophageal cancer. Tissue biopsies of Barrett’s esophagus, the premalignant condition that is linked to the development of esophageal adenocarcinoma, have displayed shortened telomeres [30], and shorter blood leukocyte telomere length among Barrett’s esophagus patients has been associated with an increased risk of future esophageal adenocarcinoma [31]. Another study suggested that chromosome-specific telomere length in blood cells may be related to esophageal cancer [32].

Given the evidence that ESCC tumor cells may have shortened telomeres and given that non-malignant esophageal epithelial cells from cancer patients have shorter telomeres compared with normal esophageal cells from non-cancer patients (suggesting a telomere-shortened epithelial field in the cancer patients) [29], we aimed to examine the telomere length of DNA extracted from balloon cytology-collected esophageal cells as a potential early detection biomarker for ESD, the histologic precursor of ESCC. These cells were collected from high-risk asymptomatic patients with a spectrum of concurrent endoscopic biopsy-proven ESD.

## Methods

### Patient population

The participants were recruited from a commune in Linxian, China, in the spring of 2002, as part of a cancer screening study using esophageal balloon cytology (Cytology Sampling Study 2), as previously described [15]. Briefly, the study targeted healthy residents aged 50- to 64-years old, although approximately 10% of the 720 participating individuals fell outside of that age range. Individuals who had any signs or symptoms of upper gastrointestinal (GI) cancer (dysphagia, hematemesis) or other chronic diseases (liver cirrhosis, congestive heart failure, unstable angina) were excluded from the study. All subjects completed a written informed consent and a short questionnaire and physical exam prior to the esophageal cancer screening procedures. The study was approved by the Institutional Review Boards of the Cancer Institute of the Chinese Academy of Medical Sciences and the U.S. National Cancer Institute.

### Balloon cytology

All subjects fasted overnight prior to the balloon cytology exam and were randomly assigned one of two esophageal balloon cytology retrieval devices, as previously described [15]. The samples used in the present study were all collected using an expandable balloon with a plastic mesh covering (Cytomesh Esophageal Cytology Device, Wilson-Cook Medical, Inc., Winston-Salem, North Carolina, USA). The patient was given 2 ml of a 2% lidocaine slurry by mouth for local anesthesia, and the balloon was inserted into the back of the throat and swallowed. Once in the stomach, the balloon and mesh covering were expanded with 7–10 mL of air and gradually withdrawn through the esophagus. The balloon, along with its collected cells from the stomach, the full length of the esophagus, and the oral cavity, was cut using sterile scissors and placed in 40 mL of saline in a 50-mL centrifuge tube, shaken, and transferred on ice to the central processing laboratory. The sample was then vortexed to remove adherent cells from the balloon. After the balloon was removed, the remaining sample was centrifuged at 1500 RPM for 5 minutes; the pellet that formed was resuspended in 1 mL of saline and snap frozen in liquid nitrogen and stored at -80°C until DNA extraction.

### Endoscopic examination

Two weeks after the balloon cytology, all subjects underwent endoscopy to examine the esophagus and stomach. After fasting overnight, the subjects were given 5 mL of a 1% lidocaine slurry by mouth for local anesthesia 2–5 minutes prior to endoscopy, which was performed using a Pentax EG-2930 or EG-2731 videoendoscope (Pentax Medical Company, Montvale, New Jersey, USA). Glycerin-free Lugol’s iodine solution was sprayed from the gastroesophageal junction to the upper esophageal sphincter. All visible lesions and Lugol’s-unstained areas in the esophagus and at least 1 normally stained midesophageal site were biopsied. The endoscopic biopsy slides were read using criteria previously described [33,34].

### DNA extraction

The Gentra Puregene Cell kit (Qiagen, Valencia, CA) was used according to the manufacturer’s instructions to extract the DNA from 300 ul of the cell suspension. The DNA quality and quantity was checked using the 260:280 ratio, Nanodrop, and Picogreen.

### Study design

We used a nested case–control design and selected subjects who had undergone both balloon cytology and endoscopy. We selected all of the subjects who had squamous dysplasia as their worst biopsy diagnosis: 38 cases of mild dysplasia, 38 cases of moderate dysplasia, and 17 cases of severe dysplasia. We then selected 94 normal controls who were matched to the squamous dysplasia cases based on age (within 5 years) and sex. In addition, we selected 50 cases of esophagitis that were matched based on age (within 5 years) and sex to the already selected controls.

### Telomere length measurement

Telomere length of the DNA samples was measured by quantitative PCR [31,35]. Each sample (200 ng) was amplified for telomeric DNA and for 36B4, a single-copy control gene, which was used as an internal control to normalize the starting amount of DNA. PCR reactions were set up with a Qiagility pipetting robot and were performed in a Rotor Gene Q (Qiagen, Valencia, CA). Samples were run in batches of 24, with each batch including 2 or 3 randomly inserted quality control samples, which came from a pool of 5 endoscopically normal subjects not selected for this study. Two additional controls were used for normalization between experiments. Periodic reproducibility experiments were performed to confirm adequate normalization. All samples, standards, and controls were run in triplicate, and the median value was used for the analyses. A standard curve was used to transform the cycle threshold into nanograms of DNA. The amount of telomeric DNA (*T*) was divided by the amount of single-copy control gene DNA (*S*), producing a relative measurement of the telomere length (*T/S* ratio). The coefficient of variation for the quantitative PCR across all batches was 8.5%.

### Covariates

The following baseline characteristics of the subjects were included in the analysis: age in years, body mass index (BMI, kg/m^2^) from measured height and weight, tobacco smoking (ever versus never), alcohol drinking (any in the past 12 months versus none), and hypertension (measured systolic blood pressure over 140 mm Hg or diastolic blood pressure over 90 mm Hg).

### Statistical analysis

Some of the selected subjects did not have sufficient DNA for telomere length measurement, so in our final analysis we had data available from 50 cases of esophagitis, 37 cases of mild dysplasia, 37 cases of moderate dysplasia, 15 cases of severe dysplasia, and 92 normal controls. Low-grade dysplasia was synonymous with mild dysplasia, while high-grade dysplasia combined the 37 cases of moderate dysplasia and 15 cases of severe dysplasia into one category. Given the number of cases and controls, we had 80% power to detect a statistically significant difference of 0.10 in telomere length between the groups.

Telomere length among the normal controls was assessed for normality, and we found no evidence for deviation from a normal distribution. Telomere length was treated as a continuous variable and as quartiles based on the distribution in controls. The Wilcoxon exact test and the analysis of variance (ANOVA) were used to compare telomere length by subject characteristics. A receiver operating characteristic (ROC) curve was plotted for the use of telomere length as a diagnostic marker for high-grade dysplasia. The association between telomere length (scaled by half of the interquartile range or as quartiles) and the worst biopsy diagnosis was assessed using unconditional logistic regression models. Adjusted models included age, sex, BMI, tobacco smoking, alcohol use, and hypertension.

All tests were two-sided, and p-values <0.05 and confidence intervals (CI) that did not overlap with 1.00 were considered statistically significant. SAS 9.2 was used for statistical analyses, and GraphPad Prism 5 was used for the ROC analysis.

## Results

The characteristics for the subjects chosen for this study are shown in Table 1. More women than men were selected. Compared with the other groups, the severe dysplasia cases had a slightly higher BMI and were more likely to smoke tobacco. In this population, those who reported smoking tobacco were almost exclusively male. Alcohol intake was relatively rare in this population. The mean telomere lengths among the normal controls (0.92) and the esophagitis (0.90) and mild (0.96), moderate (0.95), and severe dysplasia (0.97) cases were similar (p = 0.542). For further analyses, we dichotomized the dysplasia cases by categorizing the mild dysplasia cases as low-grade dysplasia and combining the moderate and severe dysplasia cases into one category of high-grade dysplasia (this combined high-grade dysplasia group had a mean telomere length of 0.96, 95% CI 0.90-1.01).

**Table 1:** Distribution of selected characteristics for the Cytology Sampling Study 2 in Linxian, China

**Characteristics**	**Normal (n = 92)**	**Esophagitis (N = 50)**	**Mild dysplasia (N = 37)**	**Moderate dysplasia (N = 37)**	**Severe dysplasia (N = 15)**
Males, N (%)	43 (47)	12 (24)	17 (46)	16 (43)	7 (47)
Age, median years (Q1-Q3)	54 (51–57)	53 (51–57)	54 (51–58)	55 (53–57)	55 (52–57)
BMI, median (Q1-Q3)	23.5 (21.5-24.8)	23.2 (21.8-25.4)	23.1 (20.8-25.2)	22.7 (20.4-24.8)	24.5 (22.5-27.2)
Ever smoke cigarettes, N (%)	28 (30)	11 (22)	10 (27)	11 (30)	5 (33)
Drink alcohol, any, N (%)	11 (12)	2 (4)	2 (5)	2 (5)	1 (7)
Hypertension, yes, N (%)	64 (68)	34 (68)	30 (81)	27 (73)	11 (73)
Telomere length^a^, mean (SD)	0.92 (0.17)	0.90 (0.16)	0.96 (0.18)	0.95 (0.17)	0.97 (0.24)
Telomere length^a^, median (Q1-Q3)^a^	0.92 (0.78-1.05)	0.88 (0.80-1.00)	0.93 (0.84-1.06)	0.94 (0.82-1.09)	0.93 (0.85-0.98)

^a^Calculated as telomeric DNA (T) divided by amount of single-copy control gene DNA (S) to produce the relative measurement of telomere length (T/S ratio).

*Abbreviations*: SD, standard deviation; Q1, first quartile; Q3, third quartile; BMI, body mass index.

Table 2 shows the distribution of telomere lengths among the controls by select subject characteristics. Telomere length did not significantly differ across any of these subject characteristics.

**Table 2:** **Telomere length**^**a **^**by selected subject characteristics among the normal controls**

**Characteristics**	**N**	**Mean**	**SD**	**Q1**	**Median**	**Q3**	**p**
Sex							
Males	43	0.91	0.17	0.78	0.92	1.03	
Females	49	0.94	0.18	0.79	0.94	1.06	0.51
Age							
<54 years	45	0.94	0.18	0.80	0.94	1.06	
≥54 years	47	0.91	0.17	0.80	0.89	1.04	0.36
BMI							
<18.5	2	0.89	-	0.83	0.89	0.95	
18.5 - <23	40	0.92	0.18	0.75	0.92	1.04	
23 - <27.5	46	0.94	0.17	0.80	0.93	1.06	
≥27.5	4	0.94	0.18	0.82	0.94	1.06	0.78
Smoke tobacco							
Ever	28	0.89	0.17	0.77	0.90	0.97	
Never	64	0.94	0.18	0.79	0.95	1.06	0.21
Drink alcohol							
Any	11	0.94	0.13	0.89	0.95	1.04	
None	81	0.92	0.18	0.78	0.91	1.05	0.65
Hypertension							
Yes	63	0.92	0.17	0.77	0.90	1.04	
No	29	0.94	0.18	0.84	0.95	1.06	0.36

^a^Calculated as telomeric DNA (T) divided by amount of single-copy control gene DNA (S) to produce the relative measurement of telomere length (T/S ratio).

*Abbreviations*: SD, standard deviation; Q1, first quartile; Q3, third quartile; BMI, body mass index.

**Figure 1:**
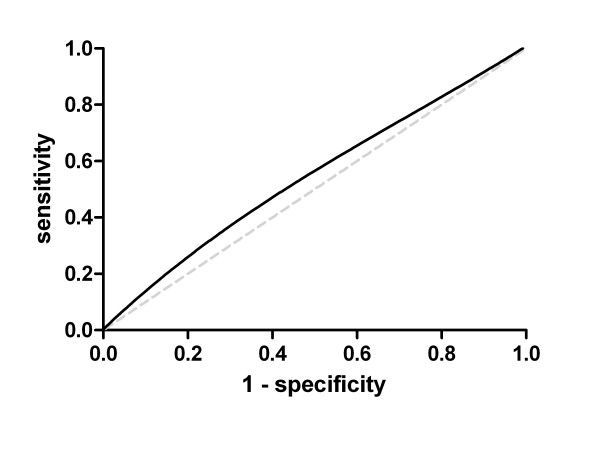
Receiver operating characteristic (ROC) curve plotted for the use of telomere length as a diagnostic marker for high-grade dysplasia (area under the curve = 0.55).

Figure 1 shows the ROC curve for the use of telomere length as a diagnostic marker for high-grade dysplasia. The area under the curve was 0.55, suggesting that telomere length of esophageal cells collected by balloon cytology is a poor marker of the presence of high-grade dysplasia.

We further assessed the association between telomere length and worst biopsy diagnosis, as shown in Table 3. Telomere length, considered either as a continuous variable or as quartiles, was not associated with esophagitis, low-grade dysplasia, or high-grade dysplasia. Adjusting for multiple potential confounders did not change the estimates.

**Table 3:** **Associations between telomere length**^**a **^**and worst biopsy diagnosis**

		**Esophagitis**		**Low-grade dysplasia**^**b**^		**High-grade dysplasia**^**c**^	
	**Controls**	**Cases**	**OR (95% CI)**	**Cases**	**OR (95% CI)**	**Cases**	**OR (95% CI)**
Unadjusted							
Continuous^d^	92	50	0.90 (0.68-1.18)	37	1.14 (0.86-1.51)	52	1.14 (0.89-1.46)
Quartiles							
<0.784	23	10	ref	4	ref	10	ref
0.784 - <0.925	23	24	2.40 (0.94-6.13)	14	3.50 (1.00-12.25)	14	1.40 (0.52-3.79)
0.925 - <1.047	23	5	0.50 (0.15-1.69)	9	2.25 (0.61-8.36)	13	1.30 (0.48-3.56)
≥1.047	23	11	1.10 (0.39-3.09)	10	2.50 (0.68-9.13)	15	1.50 (0.56-4.03)
p-trend			0.031		0.224		0.870
Adjusted^e^							
Continuous^d^	92	50	0.91 (0.68-1.21)	37	1.16 (0.87-1.54)	52	1.20 (0.92-1.56)
Quartiles							
<0.784	23	10	ref	4	ref	10	ref
0.784 - <0.925	23	24	2.46 (0.89-6.80)	14	4.80 (1.26-18.35)	14	1.51 (0.54-4.24)
0.925 - <1.047	23	5	0.59 (0.16-2.13)	9	3.07 (0.78-12.06)	13	1.50 (0.52-4.27)
≥1.047	23	11	1.08 (0.36-3.28)	10	3.05 (0.80-11.66)	15	1.83 (0.65-5.12)
p-trend			0.060		0.107		0.713

^a^Calculated as telomeric DNA (T) divided by amount of single-copy control gene DNA (S) to produce the relative measurement of telomere length (T/S ratio).

^b^Low-grade dysplasia category includes mild dysplasia cases.

^c^High-grade dysplasia category includes moderate and severe dysplasia cases.

^d^Continuous telomere length scaled by half the interquartile range based on the distribution among the normal controls.

^e^Models adjusted for age, sex, BMI, tobacco smoking, alcohol drinking, and hypertension.

*Abbreviations*: OR, odds ratio; CI, confidence interval; BMI, body mass index.

Table 4 shows the unconditional logistic regression models, both crude and adjusted, for risk of high-grade dysplasia by telomere length. Again, no associations were observed.

**Table 4:** **Association between telomere length**^**a **^**and high-grade dysplasia compared with all other diagnoses**

	**Controls and all other diagnoses**^**b**^	**High-grade dysplasia**^**c**^	
		**Cases**	**OR (95% CI)**
Unadjusted			
Continuous^d^	179	52	1.14 (0.91-1.43)
Quartiles			
<0.784	37	10	ref
0.784 - <0.925	61	14	0.85 (0.34-2.11)
0.925 - <1.047	37	13	1.30 (0.51-3.34)
≥1.047	44	15	1.26 (0.51-3.14)
p-trend			0.728
Adjusted^e^			
Continuous^d^	179	52	1.17 (0.93-1.37)
Quartiles			
<0.784	37	10	ref
0.784 - <0.925	61	14	0.93 (0.36-2.37)
0.925 - <1.047	37	13	1.43 (0.55-3.76)
≥1.047	44	15	1.39 (0.55-3.54)
p-trend			0.684

^a^Calculated as telomeric DNA (T) divided by amount of single-copy control gene DNA (S) to produce the relative measurement of telomere length (T/S ratio).

^b^Category includes normal controls, esophagitis cases, and mild dysplasia cases.

^c^High-grade dysplasia category includes moderate and severe dysplasia cases.

^d^Continuous telomere length scaled by half the interquartile range based on the distribution among the normal controls.

^e^Models adjusted for age, sex, BMI, tobacco smoking, alcohol drinking, and hypertension.

*Abbreviations*: OR, odds ratio; CI, confidence interval; BMI, body mass index.

## Discussion

ESCC is generally diagnosed at a late stage and has a very poor prognosis, so improving the methods of early detection for these cancers is both urgent and of great public health importance. We previously found that esophageal balloon cytology had low sensitivity and specificity for detecting the high-grade dysplastic lesions that are likely to progress to ESCC [14,15]. Telomere length abnormalities, which are linked to genomic stability and risk of cancer [19], have been found in ESCC [28], epithelial precursor lesions of multiple cancers [27], and, most important for the current study, in the broader non-malignant epithelial field from which squamous cell carcinomas of the esophagus arise [29]. Thus, we aimed to examine whether analysis of telomere length from esophageal balloon cytology samples could be used as an early detection screening tool. However, in this study, we found that telomere length of the esophageal cells collected by this method was not associated with the presence of low- or high-grade dysplasia in the patients, so the telomere length of such cells could not be used to identify individuals with esophageal precursor lesions who should subsequently undergo endoscopy for confirmation and treatment or follow-up of their lesions.

Most previous studies of telomere length in cancer have measured telomeres in peripheral blood lymphocytes or in cells isolated from biopsied tumors or other lesions [28-30,36,37]. By contrast, our study measured telomere length in cells collected by esophageal balloon cytology, which was a mixture of cells from the full length of the esophagus as well as some cells collected from the stomach and the oral cavity. Previously, non-neoplastic esophageal epithelial cells from ESCC patients were reported to have shorter mean telomere length than esophageal cells from non-cancer patients [29], suggesting the presence of a telomere-shortened epithelial field that could potentially be detected using balloon cytology or other analogous methods. In the current study, however, we could detect no difference in mean telomere length between participants with and without ESD. Differences between the results of our study and the previous one may reflect differences in methods, actual differences in telomere lengths in “normal” cells adjacent to ESCC and “normal” cells adjacent to ESD, and/or chance. In any case, an effective early detection biomarker in balloon cytology cell samples must be present in a broad carcinogen-altered field and must be reproducible. Thus, while we previously showed the feasibility of screening for telomerase activity in samples collected by esophageal balloon cytology [38], we demonstrate here that telomere length itself cannot serve as an early detection marker for ESD in these samples.

Our study had several limitations. We had a limited number of cases with low-grade and high-grade dysplasia. Moreover, we extracted DNA from cells collected by esophageal balloon cytology samplers, so any focal (non-field) differences in telomere length would have had to be large to be detected. Future work may examine telomere length in peripheral blood lymphocytes collected from individuals with endoscopy and biopsy-diagnosed esophageal precursor lesions.

However, our study also had several strengths, including following many of the guidelines that facilitate the development of biomarker-based screening tools suitable for early detection of cancer [39]. We used samples from a well-characterized patient population, and we included asymptomatic and apparently healthy subjects with a full spectrum of esophageal health, from normal control subjects through esophagitis and mild, moderate, and severe dysplasia. All of the subjects underwent the gold standard exam for determining esophageal health (endoscopy with Lugol’s staining and biopsy). In addition, the telomere length assay used in this study, which had a low coefficient of variation, used small amounts of DNA in a high-throughput assay and had been used in a number of previous studies of cancers in the gastrointestinal tract [23,40], including those conducted in Barrett’s esophagus and esophageal adenocarcinoma patients [31].

This is the first study to evaluate telomere length measured in esophageal balloon cytology samples as an early detection marker for esophageal precursor lesions.

## Conclusions

In conclusion, we observed no associations between telomere length in these samples and risk of low- or high-grade dysplasia, so our study provides little support for this approach.

## Abbreviations

ESD: Esophageal squamous dysplasia; ESCC: Esophageal squamous cell carcinoma; ROC: Receiver operating characteristic; GI: Gastrointestinal; BMI: Body mass index; ANOVA: Analysis of variance; CI: Confidence intervals.

## Competing interests

The authors declare that they have no competing interests.

## Authors’ contributions

SWL analyzed the data and wrote the manuscript. SWL, CCA, NDF, and SMD designed the study and interpreted the data. GM provided helpful discussion and critical reading of the manuscript. RR, DP, and PR conducted the laboratory assays. QJP, MJR, GQW, WQW, NL, PRT, and YLQ designed the sample collection and conducted the field work. All authors read and approved the final manuscript.

## Pre-publication history

The pre-publication history for this paper can be accessed here:

http://www.biomedcentral.com/1471-2407/13/578/prepub
